# The Spectrum of Pharmacological Actions of Syringetin and Its Natural Derivatives—A Summary Review

**DOI:** 10.3390/nu14235157

**Published:** 2022-12-04

**Authors:** Marcelina Chmiel, Monika Stompor-Gorący

**Affiliations:** Department of Human Pathophysiology, Institute of Medical Sciences, University of Rzeszów, Warzywna 1a, 35-310 Rzeszów, Poland

**Keywords:** syringetin, isorhamnetin, laricitrin, ayanin, pharmacological effect, anticancer dietary compounds

## Abstract

Mono- and poly-*O*-methylated flavonols and their glycoside derivatives belong to the group of natural plant polyphenols with a wide spectrum of pharmacological activities. These compounds are known for their antioxidant, antimutagenic, hepatoprotective, antidiabetic, and antilipogenic properties. Additionally, they inhibit carcinogenesis and cancer development. Having in mind the multidirectional biological activity of methylated flavonols, we would like to support further study on their health-promoting activities; in this review we summarized the most recent reports on syringetin and some of its structural analogues: laricitrin, ayanin, and isorhamnetin. Natural sources and biological potential of these substances were described based on the latest research papers.

## 1. Introduction

Polyphenolic compounds, which are commonly found in fruits, wine, and herbs, constitute a group of over 8000 substances. Among them we can find flavones and their hydroxylated analogues flavonols [1,2]. The health-promoting properties of flavonoids arise from their cyclic structures with conjugated double bonds, and from the presence of various functional groups in the aromatic rings. Methylation of free hydroxyl groups in flavonoids greatly enhances the metabolic stability and increases membrane transport, facilitating absorption and improving oral bioavailability of flavonoids [3]. Methylated and methoxylated flavones and chalcones showed potent anticancer [4,5,6], cytoprotective [7], and neuroinflammatory effects [8]. Some of them are already in the clinical trial phase [9]. Furthermore, there are studies suggesting that methylated forms of flavonoids have higher metabolic stability, bioavailability, and biological activity than non-methylated ones.

Looking at the chemical structure, syringetin is an *O*-methylated flavonol of plant origin. It is found, among others, in red wine. In the literature there are many reports on the health-promoting properties of syringetin. The compound exhibits a wide range of biological activities that include strong antioxidant, anticancer, antidiabetic, and anti-inflammatory properties and thus it may be beneficial to protect against neurodegenerative disorders such as Parkinson’s and Alzheimer’s diseases. The objective of this paper is to review the data on the possible role of syringetin in the treatment and prevention of human diseases. We summarized the current knowledge of the structure, sources, and bioactivities of syringetin and its derivatives.

### 1.1. Syringetin

Generally, methylated flavonoids demonstrate better physiological properties than non-methylated ones. Methylation of free phenolic hydroxyls provides the derivatives that are not susceptible to glucuronic acid or sulfate conjugation, which results in higher metabolic stability of the compounds. Methylation also affords much better transport of the molecules through biological membranes, for example in the intestinal absorption, and considerably enhances oral bioavailability. Moreover, methylation of flavonoids provides the derivatives with increased biological activity, e.g., having the capability to inhibit cancer cell proliferation [10].

**Figure 1:**
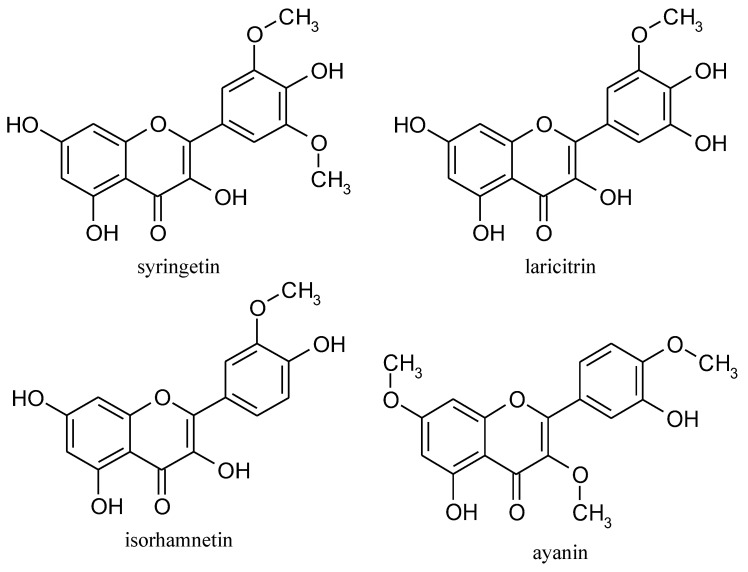
Chemical structures of syringetin, laricitrin, isorhamnetin, and ayanin.

Syringetin (3,5,7,4′-tetrahydroxy-3′,5′dimethoxyflavone) is an *O*-methylated flavonol belonging to the group of flavonoids. Structurally, it is related to several well-known phenolic compounds (Figure 1), namely laricitrin (3′-*O*-methyl derivative of myricetin), isorhamnetin, and ayanin.

Syringetin is a dimethyl myricetin derivative, which contains free hydroxyl groups at the C-2′ and C-4′ positions in ring B. It is found in red grapes, in *Lysimachia congestiflora* [11], and in *Vaccinium ashei* (blueberry) [12]. It is also one of the phenolic compounds found in wine [13,14] and in the edible parts of jambolan fruits (*Syzygium cumini* (L.) skeels) [15].

The first mention of syringetin (MW 346, C_17_H_14_O_8_) isolated from plants *Soymida febrifuga* dates back to 1972 [16]. It was first isolated as yellow needles with a m.p. of 288 °C. The extraction of the root heartwood with chloroform yielded also dihydrosyringetin (m.p. 228 °C), which does not contain a double bond between C-2 and C-3.

The presence of syringetin in the form of glucuronides was observed among others in the blueberry fruits [17]. In plants flavonoids occur most often attached to sugars. Glycosylation of flavonoids is carried out by the plant glycosyltransferase, the enzyme catalyzing the attachment of a sugar molecule into an aglycone, resulting in glycoside synthesis. Glycosides of syringetin were observed in such plants as *Caragana jubata* (Pall.) [18,19] and *Artiplex halimus* L. [20]. Syringetin-3-*O*-glucoside was also found in the skin of wine grapes such as Cabernet Sauvignon, Merlot, Syrah, and Marselan [21], needles of *Larix decidua* [22], blueberries [23], and fruits of *Embelia ribes* [24]. The presence of syringetin-3-*O*-glucoside and syringetin-3-*O*-acetylglucoside was confirmed for the first time in Cabernet Sauvignon grape and wines by Wang et al. in 2003 [25].

Moreover, syringetin and its isomer (2R, 3R)-dihydrosyringetin were found in the seeds of *Hovenia dulcis*, the plant used in traditional Chinese medicine to relieve thirst [26]; in *Vitis vinifera* grapes and wines [14,27]; and in *Abies amabilis* [28]. In addition, the other valuable source of syringetin and its derivatives with antioxidant and antimicrobial properties are residues of the wine industry, including grape pomace [29]. Syringetin-3-glucoside and dihydroquercetin-3-hexoside are the characteristic flavonols of red and white wines, respectively, fabricated from spine grape (*Vitis davidii* Foex) [30]. As the aglycone, the compound was found for example in the leaves of *Cedrus atlantica glauca* [31] and in petal and leaf-stem of *Limnanthes douglasii* [32]. Williams and Harborne found syringetin in Zingiberales where its content was about 3%. Moreover, a new glycoside, syringetin-3-rhamnoside was identified in the leaves of *Hedychium stenopetalum* by the same researchers [33]. Syringetin-3-rutinoside and laricitrin-3-glucoside were found by Tyukavkina et al. [34] in the needles of *Larix sibirica*; whereas methanolic extract of *Anthyllis sericea* contained syringetin-3-galactoside [35]. Similarly, syringetin in the form of galactoside was observed in *Lysimachia vulgaris var davurica* together with astragalin [36]. Extraction of whole plant of *Lysimachia nummularia* apart from syringetin-3-galactoside led to isolation of a new derivative, syringetin-3-*O*-xylopyranoside [37]. The research group of Mizuno et al. [38] isolated syringetin-3-[6″-acetylglucosyl(1-3)galactoside] from the underground parts of *Achlys triphylla*. Syringetin-3-*O*-(6″-acetyl)-β-glucopyranoside was isolated from needles of Norway spruce (*Picea abies*) [39]. In a later study carried out by Slimestad and Hostettmann [40] another syringetin derivative was identified in this plant, namely syringetin-3-*O*-rutinoside. Additionally, a new flavonol, syringetin-6-C-glucoside, was isolated by Wu et al. [41] from *Moghania macrophylla*. Syringetin-3-*O*-α-arabinofuranoside and syringetin-3-rhamnoside were isolated from the whole plant of *Lysimachia congestiflora* [13]. Syringetin-3-*O*-robinobioside was identified by Brun et al. [42] in *Catharanthus roseus*; whereas syringetin-3-*O*-α-rhamnopyranoside was isolated from the methanol extract of the fruit of rabbiteye blueberry *Vaccinium ashei* [12]. Moreover, syringetin-3-O-β-glucopyranoside and syringetin-3-*O*-β-galactopyranoside were isolated from the fruit of *Vaccinium uliginosum* and *Vaccinium myrtillus* [43,44], while syringetin-*O*-hexoside was identified in *Aquilaria malaccensis* by Eissa et al. [45].

Syringetin was identified also in various types of Tunisian figs belonging to the Smyrna-type *Ficus carica* varieties known as Kholi, Tchich Asal, Himri, and Bidhi [46].

Glycoside derivatives of syringetin may be also obtained as a result of biotransformation of myricetin in the culture of *P. americana* cells. According to the study by Fujitaka et al. [47] myricetin was glucosylated, then methylation of the glucosylated product at 3′- and 5′ positions occurred in a 2-day biotransformation process to provide syringetin-3-*O*-β-d-glucoside.

### 1.2. Pharmacological Effects of Syringetin

*O*-Methylated flavonols attract a great interest within the research community as active substances with anticancer potential [48,49]. Anticancerogenic properties of these compounds arise from their antioxidant properties, their ability to change activities of some enzymes and to modulate certain biosynthetic pathways [50,51,52]. According to the research by Gómez-Alonso et al. [53], syringetin alone as well as a mixture of flavonols composed of quercetin, myricetin, laricitrin, and syringetin (45:35:10:10) are capable to inhibit the proliferation of colorectal epithelial adenocarcinoma cells Caco-2 (ECACC). An exposure to syringetin (50 µM) induced a dose-dependent reduction in cyclin D1 and COX-2 levels, which is up-regulated in many cancers. Syringetin is capable to inhibit the growth of cancer cells both via the induction of cell cycle arrest in the G_2_/M phase and the initiation of apoptosis. In the cells exposed to syringetin, there was a 16.7% reduction in the proportion of cells in G_0_/G_1_ and a 16.1% increase in the proportion in G_2_/M.

There are some hypotheses that syringetin may be used to prevent bone metastasis in patients with lung cancer. Tsai et al. [54] reported that syringetin may suppress in vitro osteoclastogenesis mediated by osteoblasts in human lung adenocarcinoma (A549 and CL1-5) in a dose-dependent manner. Induction of differentiation by syringetin is associated with the increased activation of SMAD1/5/8 and extracellular signal-regulated kinase 1/2 (ERK1/2).

Bando et al. [55] hypothesized that syringetin can be employed for the development of novel effective radiosensitizers. The study confirmed that syringetin effectively enhanced radiosensitivity of cancer cells (H1299 and C3H/MCA clone 15) compared with normal ones (HFL-III and C3H/10T1/2), through enhancement of the caspase-3-mediated apoptosis pathway.

Among the substances of plant origin there is a search for promising compounds that would help in prevention and treatment of metabolic diseases that negatively affect digestion and absorption of nutrients, e.g., carbohydrates. The effect of flavonoids on the cellular metabolism of lipids is a good reason to use them as dietary therapeutic agents in cancer treatment [56]. Many plant flavonoids demonstrate antidiabetic properties [57]. The most promising substances are subjected to clinical trials [58]. According to Wu et al. [59] syringetin belongs to alpha-glucosidase (AGH) inhibitors; therefore, in the future it may be used in the treatment of diabetes so as to inhibit absorption of carbohydrates and reduce postprandial glycemia. The established IC_50_ value for syringetin (36.8 µM) was lower than for the antidiabetic drug acarbose, which was used as a positive control. Moreover, syringetin and isorhamnetin bound to hexose (syringetin 3-*O*-hexoside and isorhamnetin 3-*O*-hexoside) are natural ingredients of *Cercis chinensis* Bunge fruits, contributing to their high α-glucosidase inhibitory activity (IC_50_ = 11.94 ± 1.23 μg/mL), higher than for acarbose [60]. There is a hypothesis that syringetin 3-*O*-hexoside, being an ingredient of *Lilium* species, is responsible for high antioxidant activity of methanolic extracts of their bulbs [61].

Whereas, Lau and Chang [62] demonstrated that syringetin is not an activator of mouse pregnane X receptors (PXR, NR1I2), which regulate the expression of selected genes involved in drug transport and biotransformation. Syringetin-3-*O*-β-d-glucoside showed low DPPH and ABTS radical scavenging activity, with IC_50_ values of 286.6 ± 3.5 for DPPH and 283.0 ± 1.5 μg/mL for ABTS [63].

According to the literature, methylated flavonoids may modulate aging- or stress-related pathways in the nematode. Büchter et al. [64] documented that syringetin enhanced the life span of *C. elegans* by 35.7%. They also analyzed the effect of methylated derivatives, including syringetin, on the accumulation of lipofuscin, which is the autofluorescent age pigment, consisting of molecular aggregates of highly oxidized proteins and lipids. Syringetin caused even stronger reduction (46.1%) in the lipofuscin fluorescence than myricetin (33.1%).

The studies by Kakorin’s team [18] showed that the lyophilized aqueous extract of *Caragana jubata*, containing mono- and diglycosides of *O*-methylated flavonols (syringetin and syringetin-3-rhamnoside) had moderate antimicrobial activity against pathogenic microorganisms, such as Gram-positive (*Staphylococcus aureus* 209-P ATCC 6538) and Gram-negative bacteria (*Escherichia coli* ATCC 25922, *Proteus vulgaris* ATCC 6896, *Pseudomonas aeruginosa* ATCC 9027), yeasts (*Candida albicans* ATCC 10231), and mycelial fungi (*Microsporum canis* 352) at the concentration from 4.000 µg/ml to 4.500 µg/mL. Earlier studies by the same research team [19] showed that the content of phenolic compounds was stable during the long-term storage of the dried raw material.

Furthermore, El-Asar et al. [20] isolated two *O*-methylated flavonols from the aerial parts of *Atriplex halimus* L. They were identified as syringetin 3-*O*-β-d-rutinoside and syringetin 3-*O*-β-d-glucopyranoside, which may act as immunomodulators. The results of the anti-inflammatory studies showed that both compounds markedly increased the levels of cytokines and pro-inflammatory mediators. What is more, the glucopyranoside derivatives possessed also good antimicrobial activity with inhibition zone diameter from 10 mm for *Ac. baumanii* to 19 mm for *S. pyogenes*. The zone diameter of 17 mm was observed for *S. aureus*, and *E. faecalis*. 3-*O*-β-d-rutinoside was active against *S. aureus*, *S. pyogenes*, *E. faecalis,* and *C. albicans*, with the inhibition zone from 10 mm to 15 mm. Syringetin 3-*O*-β-d-glucopyranoside was identified also in fresh leaves of *Eucalyptus maideni* [65].

Flavonoids, including methoxy derivatives of 3-hydroxyflavonol have also antiviral properties. The anti- respiratory syncytial virus (RSV) activity of syringetin isolated from *Hovenia dulcis* was reported for the first time by Xu et al. [26]. The study was performed using the RSV A2 strains, which were cultivated in HEp-2 cells. The substances were tested using the cytopathic effect reduction assay (CPE) in comparison with the positive control ribavirin (IC_50_ = 51.23 ± 10.25 µM).

The best results were noted for two structural analogues of syringetin: kaempferol (IC_50_ = 64.69 ± 5.24 µM) and myricetin (IC_50_ = 130.21 ± 8.68 µM). The substituents, such as hydrogen, hydroxyl, and methoxy groups in the B ring of the compounds affected their anti-RSV activities. Syringetin was inactive in this assay. The 50% cytotoxic concentration (CC_50_) calculated as the concentration of a tested compound required to reduce cell viability by 50% (compared with untreated cells, incubated with the medium only) was estimated through the OD values and for syringetin it was over 100 µM.

Grewal et al. [66] subjected syringetin to molecular docking studies in order to investigate its binding interactions with eight anti-Alzheimer’s drug targets. In the in silico study syringetin showed strong binding interactions and complementary orientation pattern in the binding site of all the targets involved in pathogenesis of AD. The evaluated parameters included molecular weight (MW), distribution coefficient (log D), partition coefficient (log P), water solubility (log Sw), topological polar surface area (tPSA), hydrogen bond donors (HBDs), hydrogen bond acceptors (HBAs), solubility (mg/L), and number of rotatable bonds (NRBs).

In addition, another research revealed that some other flavonoid compounds, such as non-methylated syringetin analogues, are beneficial for the treatment of Alzheimer’s disease. As it was described by Ramezani et al. [67], myricetin protects hippocampal CA3 pyramidal neurons and improves learning and memory impairments in rats with Alzheimer’s disease.

It has been reported that certain flavonoids are activators of the rat vitamin D receptor (rVDR), which except for acting as the major regulator of vitamin D and calcium homeostasis, controls also the expression of genes involved in the transport, bioactivation, and detoxification of endogenous substances and xenobiotics, including drugs. As it was demonstrated by Lau et al. [68], syringetin is not an agonist of rat vitamin D receptor (rVDR), mouse VDR (mVDR), or human VDR (hVDR), as judged by cell-based and in silico evidence. Syringetin does not activate the rVDR, mVDR, or hVDR in HEK-293 and HepG2 cells transfected with the corresponding receptor expression plasmid and either the secreted phosphoprotein 1 (Spp1) or cytochrome P450 24A1 (CYP24A1) reporter plasmid compared with the respective empty vector control group transfected with one or the other reporter plasmid and treated with tested flavonol.

Due to the correlation between the endocrine and the osteoarticular systems, interactions of methylated flavonols and their impact on bone formation were investigated. Hsu et al. [69] demonstrated that syringetin significantly induces differentiation in MC3T3-E1 mouse calvaria osteoblasts and in human fetal osteoblastic 1.19 cell line. The results indicate that syringetin stimulates osteoblast differentiation at various stages, from maturation to terminally differentiated osteoblasts. Induction of differentiation by syringetin is associated with increased bone morphogenetic protein-2 (BMP-2) production and increased activation of SMAD1/5/8 and extracellular signal-regulated kinase 1/2 (ERK1/2).

The research on methylated myricetin derivatives (laricitrin, syringetin, and myricetin trimethyl ether) revealed that these compounds strongly enhance the life span of *C. elegans* and increase the stress resistance of the nematode. The pro-longevity effect was dependent on DAF-16. 

Büchter et al. [64] used methylated derivatives of myricetin (laricitrin and syringetin) to examine whether the free OH groups in ring B are necessary for the life span extending effect. Such as in the case of myricetin, all the methylated derivatives extended the life span, reduced oxidative stress (DCF), and reduced the accumulation of lipofuscin. In contrast to myricetin, the methylated derivatives strongly increased the resistance against thermal stress. What is more, treatment with the methylated compounds induced a much stronger nuclear localization of the DAF-16 transcription factor (FoxO homologue).

### 1.3. Laricitrin

One of the structural analogues of syringetin is the monomethylated myricetin named laricitrin. The compound also possesses an interesting biological activity. It is a dietary flavonoid derivative, which can be found for example in grapes, red wine, *Rhododendron luteum* [70], *Carpobrotus edulis* [71], leaves of *Psidum litorale* [72], *Ginkgo biloba* [73], and needles *of Larix sibirica* Ledb. The content of laricitrin and other health-promoting compounds in vine can be increased by means of malolactic fermentation (MLF) with the help of recombinant *Pediococcus acidilactici* BD16 (fsc+/ech+) strains [74].

Chang et al. [75] documented that laricitrin inhibited progression of lung cancer cells induced by benzo(a)pyrene (BaP) in the lung cancer tumor microenvironment. In the study, human lung adenocarcinoma cell lines (H1395, H1975, H2087, and HCC2935) were used. Laricitrin can be also an efficacious immunoadjuvant and has a synergistic effect when combined with chemotherapy. This compound potentiated the anticancer activity of cisplatin in mouse models [76]. Being an ingredient of aqueous extracts of *Asparagus officinalis* L., laricitrin-*O*-glucoside contributes to its high antitumor activity against breast cancer cells (NIH/3T3, MDA-MB-231, and MCF-7). The most sensitive to laricitrin proved MCF-7 cell line (expressing HR^+^/HER2^−^ phenotype), where a significant reduction in proliferation was observed, along with the cell cycle arrest and low levels of apoptosis [77]. Anticancer activity of laricitrin may be associated among others with inhibition of the breast cancer resistance protein (BCRP/ABCG2) [78].

### 1.4. Isorhamnetin

According to Mattivi et al. [79], isorhamnetin is one of the main flavonols contained in grapes. In red grapes the reported content of this compound was 3.89%, whereas in white ones the reported content was 1.74%. Interestingly, laricitrin and syringetin were missing in all white varieties, indicating that the enzyme flavonoid 3‘,5‘-hydroxylase is not expressed in white grape varieties. In red grapes the content of 3.22% and 5.65% was determined for syringetin and laricitrin, respectively. Isorhamnetin in the form of rutoside (isorhamnetin-3-*O*-rutinoside) is found in beverages with yerba mate (*Ilex paraguariensis*). It has been proved that being an ingredient of drinks, yerba mate inhibits fat absorption in vivo by inhibition of pancreatic lipase activity. Therefore, it may be used to stabilize body weight and the overall lipid metabolism [80]. A new derivative of isorhamnetin, namely isorhamnetin-3-*O*-(2”-*O*-galloyl)-β-d-glucopyranoside, was identified in the petals of *R. rugosa*, which are used in various cosmetic products [81]; whereas isorhamnetin glycosides found in extracts of various Spiraea species (*S. media*, *S. hypericifolia*, *S. salicifolia* L.) in high probability are responsible for the antiviral activity of these extracts against viruses of influenza A and B [82]. Glycoside derivatives of isorhamnetin (isorhamnetin-3-O-β-d-glucoside) were prepared by biotransformation of quercetin in the culture of *Phytolacca americana* [47]. In turn, the stepwise bioconversion of rutin by *Eurotium amstelodami* BSX001 leads to aglycones such as quercetin, kaempferol, and isorhamnetin [83]. Moreover, isorhamnetin is one of the main ingredients of a common food seasoning *Zanthoxylum schinifolium*, known for its special aroma [84], and in fermented vegetables such as pickled chayote [85]. An in vitro study showed that isorhamnetin may be used as a potential therapeutic compound against COVID-19 in the future [86].

The most recent literature reports indicate that being an ingredient of a novel drug Bushao Tiaozhi Capsule (BSTZC), isorhamnetin may help to regulate the lipids level in the blood, and therefore may be used in the treatment of hyperlipidemia [87]. In addition, an in vivo study on rats showed that isorhamnetin delivered as one of the ingredients of Corinthian currant (dried fruits) easily comes across the blood–brain barrier [88].

### 1.5. Ayanin

Another syringetin derivative is ayanin, being 3,7,4′-tri-*O*-methylated derivative of quercetin, which can be found among others in *Croton schiedeanus*, *Nothofagus gunnii* [89], and *Psiadia trinervia* [90]. As an aglycone it was identified in *Ageratina deltoidea* [91], leaves of *Larrea nitida* Cav. [92], and leaves of *Nothofagus cunninghami* [89]. Ayanin 3′-O-β-d-glucopyranoside was isolated also from aerial parts of *Dasiphora parvifolia* [93].

The low-density lipoprotein (LDL)-antioxidant activity of ayanin isolated from leaves and stems of *Plectranthus hadiensis var. tomentosus* was reported with the IC_50_ = 53.7 μM by Ji et al. [94]. In addition, Ahn et al. [82] revealed that ayanin markedly down-regulated the PMA+A23187-induced synthesis of interleukin-6 (IL-6) in HMC-1 cells without cytotoxicity, with the IC_50_ value of 17.8 µM (montelukast served as a positive control with the IC_50_ = 8.7 µM).

Also, the analogue of ayanin, 3,4′,7-*O*-trimethylquercetin, possess interesting biological properties. Yamauchi et al. [95] reported that it may inhibit ovarian cancer cell (SKOV-3) migration and invasion without effecting proliferation. Furthermore, this compound inhibited the expression of urokinase plasminogen activator (uPA) and MMP-2 matrix metalloproteinase (MMP-2) but it had no effect on plasminogen activator inhibitor 1 (PAI-1) and proliferating cell nuclear antigen (PCNA).

Moreover, ayanin was found to be a highly potent inhibitor of breast cancer resistance protein (BCRP) encoded by the ABCG2 gene, showing only slightly lower activity than Ko143, the most potent ABCG2 inhibitor known so far [96]. The authors hypothesized that consumption of flavonoids with high inhibitory activity can change pharmacokinetics and levels of drugs that are BCRP substrates. The exchange of the 3-methoxy group for a hydroxyl one, acting also as a hydrogen bond donor, resulted in a decrease in the activity, underlining the potential role of the hydrogen bond acceptor 3-OCH_3_ for the interaction with BCRP.

To sum, mono- and poly-O-methylated flavonols and their glycoside derivatives belonging to the group of natural plant polyphenols may be used to treat various diseases as natural antioxidants, anti-inflammatory, or anticancer substances. Unfortunately, biological activity of many glycosylated methylflavonols, including syringetin derivatives, have not been studied so far. Similarly, the metabolism of syringetin in microorganisms is not well-known. Molecular and biochemical mechanisms of action of syringetin and its derivatives also require further investigation, as well as their effect on the enzymes that are responsible for maintaining homeostasis in the human body. Such studies may lead to promising preclinical and clinical research results, and may contribute to development of innovative and safe prodrugs based on the structure of syringetin.

The preclinical data collected in this article unequivocally indicate that syringetin has health-promoting properties. The mechanisms of its pharmacological actions are related to inhibition of cancer cell proliferation, modulatory effects on the key enzymes responsible for development of metabolic diseases, and the enzymes responsible for maintaining homeostasis in the human body and interactions with cell receptors. Syringetin may have beneficial effect on alleviation of side effects associated with the oncological treatment, chemo- and radiotherapy. According to the current state of knowledge, no negative effects of syringetin have been noticed. Nevertheless, despite many studies that have been carried out so far, further in vitro and in vivo analyses are needed, and most of all, syringetin should be subjected to clinical trials so as to assess its direct effects on human organism. These allow the safe use of syringetin in medicine.

## 2. Conclusions

Methoxylated flavonoids are widely found in many plants, including fruits, vegetables, or seeds, where they are usually bound to sugars. Depending on the chemical structure of the substrates, the nature and positions of substituents in the aromatic rings, the number and nature of glycosidic bonds, and finally the presence of both free and methylated hydroxyl groups, they have different pharmacological effects. They may be used to treat various diseases as natural antioxidants, anti-inflammatory, or anticancer substances. Unfortunately, biological activity of many glycosylated methylflavonols, including syringetin derivatives, have not been studied so far. Similarly, the metabolism of syringetin in microorganisms is not well-known. Molecular and biochemical mechanisms of action of syringetin and its derivatives also require further investigation, as well as their effect on the enzymes that are responsible for maintaining homeostasis in the human body. Such studies may lead to promising preclinical and clinical research results, and may contribute to development of innovative and safe prodrugs based on the structure of syringetin.

## Data Availability

Data is contained within the article.

## References

[1] Stompor-Gorący M., Bajek-Bil A., Machaczka M. (2021). Chrysin: Perspectives on contemporary status and future possibilities as pro-health agent. Nutrients.

[2] Panek-Krzyśko A., Stompor-Gorący M. (2021). The pro-health benefits of morusin administration–an update review. Nutrients.

[3] Wen X., Walle T. (2006). Methylated flavonoids have greatly improved intestinal absorption and metabolic stability. Drug Metab. Dispos..

[4] Stompor M. (2020). A review on sources and pharmacological aspects of sakuranetin. Nutrients.

[5] Park C., Cha H.J., Choi E.O., Lee H., Lee H., Hwang-Bo H., Ji S.Y., Kim M.Y., Kim S.Y., Hong S.H. (2019). Isorhamnetin induces cell cycle arrest and apoptosis via reactive oxygen species-mediated AMP-activated protein kinase signaling pathway activation in human bladder cancer cells. Cancers.

[6] Ming-Yu H., Ming-Ju H., Yu-Sheng L., Chia-Chieh L., Yi-Ching C., Mu-Kuan C., Ming-Chih C. (2022). Xanthohumol target the JNK1/2 signaling pathway in apoptosis of human nasopharyngeal carcinoma cells. Environ. Toxicol..

[7] Zhou W., Yang L., Deng K., Xu G., Wang Y., Ni Q., Zhang Y. (2023). Investigation of isoflavone constituents from tuber of Apios americana Medik and its protective effect against oxidative damage on RIN-m5F cells. Food Chem..

[8] Lee D.S., Lee M., Sung S.H., Jeong G.S. (2016). Involvement of heme oxygenase-1 induction in the cytoprotective and neuroinflammatory activities of *Siegesbeckia pubescens* isolated from 5,3′-dihydroxy-3,7,4′-trimethoxyflavone in HT22 cells and BV2 cells. Int. Immunopharmacol..

[9] Langley B.O., Ryan J.J., Phipps J., Buttolph L., Bray B., Aslan J. (2022). E Xanthohumol microbiome and signature in adults with Crohn’s disease (the XMaS trial): A protocol for a phase II triple-masked, plabeco-controlled clinical trial. Trials.

[10] Möller G., Temml V., Cala Peralta A., Gruet O., Richomme P., Séraphin D., Viault G., Kraus L., Huber-Cantonati P., Schopfhauser E. (2022). Analogueues of natural chalcones as efficient inhibitors of AKR1C3. Metabolites.

[11] Guo J., Yu D.L., Xu L., Zhu M., Yang S.L. (1998). Flavonol glycosides from *Lysimachia congestiflora*. Phytochemistry.

[12] Ono M., Koto M., Komatsu H., Igoshi K., Kobayashi H., Ito Y., Nohara T. (2004). Cytotoxic triterpenes and sterol from the fruit of rabbiteye blueberry (*Vaccinium ashei*). Food Sci. Technol. Res..

[13] Favre G., González-Neves G., Piccardo D., Gómez-Alonso S., Pèrez-Navarro J., Hermosín-Gutièrrez I. (2018). New acylated flavonols identified in *Vitis vinifera* grapes and wines. Food Res. J..

[14] Zhu L., Zhang Y., Lu J. (2012). Phenolic contents and compositions in skins of red wine grape cultivars among various genetic backgrounds and orginations. Int. J. Mol. Sci..

[15] Tavares I.M.C., Lago-Vanzela E.S., Rebello L.P.G., Ramos A.M., Gómez-Alonso S., García-Romero E., Da-Silva R., Hermosín-Gutièrrez I. (2016). Comprehensive study of the phenolic composition of the edible parts of jambolan fruits (*Syzygium cumini* (L.) skeels. Food Res. Int..

[16] Pardhasaradhi M., Sidhu G.S. (1972). Obtusifoliol, syringetin and dihydrosyringetin from Soymida febrifuga. Phytochemistry.

[17] Pertuzatti B., Teixeira Barcia M., Gómez-Alonso S., Teixeira Godoy H., Hermosin-Gutierrez I. (2021). Phenolics profiling by HPLC-DAD-ESI-MSn aided by principal component analysis to classify rabbiteye and Highbush blueberries. Food Chem..

[18] Kakorin P.A., Fateeva T.V., Tereshkina O.I., Perova I.B., Ramenskaya G.V., Sologova S.S., Eller K.I. (2020). Antimicrobial activity of liophillized aqueous extract from *Caragana jubata* (Pall.) poir. Pharm. Chem. J..

[19] Kakorin P.A., Babenkova I.V., Teselkin Y.P., Ramenskaya G.V., Demura T.A., Kukes V.G. (2018). Hepatoprotective activity of aqueous extract from *Caragana jubata* (Pall.) Poir shoots in the model of acute hepatitis induced by acetaminophen in rats. Biomed. Khim..

[20] El-Aasr M., Kabbash A., Abo El-Seoud K.A., Al-Madboly L.A., Ikeda T. (2016). Antimicrobial and immunomodulatory activities of flavonol glycosides isolated from *Atriplex halimus* L. herb. J. Pharm. Sci. Res..

[21] Shi P.B., Yue T.X., Ai L.L., Cheng Y.F., Meng J.F., Li M.H., Zhang Z.W. (2016). Phenolic compound profiles in grape skins of Cabernet Sauvignon, Merlot, Syrah and Marselan cultivated in the Shachen area (China). S. Afr. Enol. Vitic..

[22] Niemann G.J., Baas W.J. (1978). Phenolics from larix needles XIV flavonoids and phenolic glucosides and ester of *L. decidua*. Z. Nat. C J. Biosci..

[23] Pico J., Yanm Y., Gerbrandt E.M., Castellarin S.D. (2022). Determination of free and bound phenolic in northern highbush blueberries by a validated HPLC/QTOF methodology. J. Food Compos. Anal..

[24] Qin Y., Chen J.P., Li C.Y., Zhu L.J., Zhang X., Wang J.H., Yao X.S. (2021). Flavonoid glycosides from the fruits of Embelia ribes and their anti-oxidant and α-glucosidase inhibitory activities. J. Asian Nat. Prod. Res..

[25] Wang H., Race E.J., Shikhande A.J. (2003). Anthocyanin transformation in Cabernet Sauvignon wine during aging. J. Agric. Food Chem..

[26] Xu F., Gao M., Li H., Han X., Zhang X., Li Y., Guo D., Liu B. (2020). Three new bisflavonols from the seeds of *Hovenia dulcis* Thunb. and their anti-RSV activities. Fitoterapia.

[27] Ferreira V., Fernandes F., Pinto-Carnide O., Valentão P., Falco V., Martín J.P., Ortiz J.M., Arroyo-García R., Andrade P.B., Castro I. (2016). Identification of *Vitis vinifera* L. grape berry skin color mutants and polyphenolic profile. Food Chem..

[28] Parker W.H., Maze J., McLachlan D.G. (1979). Flavonoids of *Abies amabilis* needles. Phytochemistry.

[29] Peixoto C.M., Dias M.I., Alves M.J., Calhelha R.C., Barros L., Pinho S.P., Ferreira I.C.F.R. (2018). Grape pomace as a source of phenolic compounds and diverse bioactive properties. Food Chem..

[30] Meng J.F., Xu T.F., Qin M.Y., Zhuang X.F., Fang Y.L., Zhang Z.W. (2012). Phenolic characterization of young wines made from spine grape (*Vitis davidii Foex*) grown in Chongyi County (China). Food Res. Int..

[31] Niemann G.J. (1977). Flavonoids and related compounds in leaves of Ponaceae. II. *Cedrus atlantica* c.v. glauca. Z. Nat. C J. Biosci..

[32] Parker W.H., Bohm B.A. (1975). Flavonol glycosides of *Limnanthes douglasii*. Phytochemistry.

[33] Williams C.A., Harborne J.B. (1977). The leaf flavonoids of the *Zingiberales*. Biochem. Syst. Ecol..

[34] Tyukavkina N.A., Medvedeva S.A., Ivanova S.Z. (1974). New flavonol glycosides from the needles of *Larix sibirica*. Chem. Nat. Compd..

[35] Adell J., Barbera Q., Alberto Marco J. (1988). Flavonoid glycosides from *Anthyllis sericea*. Phytochemistry.

[36] Yusukawa K., Takido M. (1988). Quercetin 3-rhamnosyl (1 → 2) galactoside from *Lysimachia vulgaris* var. *davurica*. Phytochemistry.

[37] Yasukawa K., Ogawa H., Takido M. (1990). Two flavonol glycosides from *Lysimachia nummularia*. Phytochemistry.

[38] Mizuno M., Yoshida S., Linuma M., Tanaka T., Tsuji K., Lang F.A. (1992). Four flavonol glycosides from *Achlys triphylla*. Phytochemistry.

[39] Slimestad R., Andersen Q.M., Francis G.W., Marston A., Hostettmann K. (1995). Syringetin 3-O-(6″-acetyl)-β-glucopyranoside and other flavonols from needles of norway spruce, *Picea abies*. Phytochemistry.

[40] Slimestad R., Hostettmann K. (1996). Characterisation of phenolic constituents from juvenile and mature needles of Norway spruce by means of high performance liquid chromatography-mass spectrometry. Phytochem. Anal..

[41] Wu J.B., Cheng Y.D., Su L.L., Kuo C.W., Kuo S.C. (1997). A flavonol C-glycoside from *Moghania macrophylla*. Phytochemistry.

[42] Brun G., Dijoux M.G., David B., Mariotte A.M. (1998). A new flavonol glycoside from *Catharanthus roseus*. Phytochemistry.

[43] Liu S., Marsol-Vall A., Laaksonen O., Kortesniemi M., Yang B. (2020). Characterization and quantification of nonanthocyanin phenolic compounds in white and blue bilberry (*Vaccinium myrtillus*) juices and wines using UHPLC-DAD-ESI-QTOF-MS and UHPLC-DAD. J. Agric. Food Chem..

[44] Masuoka C., Yokoi K., Komatsu H., Kinjo J., Nohara T., Ono M. (2007). Two novel antioxidant ortho-benzoyloxyphenyl acetic acid derivatives from the fruit of *Vaccinium uliginosum*. Food Sci. Technol. Res..

[45] Eissa M.A., Hashim Y.Z.H.Y., El-Kersh D.M., Abd-Azziz S.S.S., Salleh H.M., Isa M.L.M., Abd Warif N.M. (2020). Metabolite profiling of *Aquilaria malaccensis* leaf extract using liquid chromatography-Q-TOF-mass spectrometry and investigation of its potential antilipoxygenase activity in vitro. Processes.

[46] Soltana H., De Rosso M., Lazreg H., Dalla Vedova A., Hammami M., Flamini R. (2018). LC-QTOF characterization of non-anthocyanic flavonoids in four Tunisian fig varieties. J. Mass Spectrom..

[47] Fujitaka Y., Shimoda K., Kubota N., Araki M., Onishi T., Nakayama N., Ishihara K., Tanigawa M., Hamada H., Hamada H. (2017). Glycosylation and methylation of quercetin and myricetin by cultured cells of *Phytolacca amerykany*. Nat. Prod. Commun..

[48] Stompor M., Uram Ł., Podgórski R. (2017). In vitro effect of 8-prenylnaringenin and naringenin on fibroblasts and glioblastoma cells-cellular accumulation and cytotoxicity. Molecules.

[49] Stompor M., Świtalska M., Wietrzyk J. (2019). The influence of a single and double biotinylation of xanthohumol on its anticancer activity. Acta Biochim. Pol..

[50] Yen S.C., Wu Y.W., Huang C.C., Chao M.W., Tu H.J., Chen L.C., Lin T.E., Sung T.Y., Tseng H.J., Chu J.C. (2022). *O*-Methylated flavonol as multi-kinase inhibitor of leukemogenic kinases exhibits a potential treatment for acute myeloid leukemia. Phytomedicine.

[51] Vetrivel P., Kim S.M., Ha S.E., Kim H.H., Bhosale P.B., Senthil K., Kim G.S. (2020). Compound prunetin induces cell death in gastric cancer cell with potent anti-proliferative properties: In vitro assay, molecular docking, dynamics, and admet studies. Biomolecules.

[52] Park S., Bazer F.W., Lim W., Song G. (2018). The *O*-methylated isoflavone, formononetin, inhibits human ovarian cancer cell proliferation by sub G_0_/G_1_ cell phase arrest through PI3K/AKT and ERK1/2 inactivation. J. Cell. Biochem..

[53] Gómez-Alonso S., Collins V.J., Vauzour D., Rodríguez-Mateos A., Corona G., Spencer J.P.E. (2012). Inhibition of colon adenocarcinoma cell proliferation by flavonols is linked to a G_2_/M cell cycle block and reduction in cyclin D1 expression. Food Chem..

[54] Tsai Y.M., Chong I.W., Hung J.Y., Chang W.A., Kuo P.L., Tsai M.J., Hsu Y.L. (2015). Syringetin suppresses osteoclastogenesis mediated by osteoblasts in human lung adenocarcinoma. Oncol. Res..

[55] Bando S.I., Hatano O., Takemori H., Kubota N., Ohnishi K. (2017). Potentiality of syringetin for preferential radiosensitization to cancer cells. Int. J. Radiat. Biol..

[56] Rosa A., Isola R., Pollastro F., Caria P., Appendino G., Nieddu M. (2020). The dietary flavonoid eupatilin attenuates in vitro lipid peroxidation and targetes lipid profile in cancer Hela cells. Food Funct..

[57] Lim S.H., Yu J.S., Lee H.S., Choi C.I., Kim K.H. (2021). Antidiabetic flavonoids from fruits of *Morus alba* promoting insulin-stimulated glucose uptake via akt and AMP-activated protein kinase activation in 3T3-L1 adipocytes. Pharmaceutics.

[58] Naeini F., Namkhah Z., Tutunchi H., Rezayat S.M., Mansouri S., Jazayeri-Tehrani S.A., Yaseri M., Hosseizadeh-Attar M.J. (2021). Effects of naringenina supplementation in overweight/obese patients with non-alcoholic fatty liver disease: Study protocol for a randomized double-blind clinical trial. Trials.

[59] Wu B., Song H.P., Zhou X., Liu X.G., Gao W., Dong X., Li H.J., Li P., Yang H. (2016). Screening of minor bioactive compounds from herbal medicines by in silico docking and the trace peak exposure methods. J. Chromatogr. A.

[60] Hong Y., Liao X., Chen Z. (2022). Screening and charazterization of potential α-glucosidase inhibitors from Cercis chinensis Bunge fruits using ultrafiltration coupled with HPLC-ESI-MS/MS. Food Chem..

[61] Xu Q., Zechen S., Long Y., Zhang L., Pan Y., Li Q. (2022). Analyses on antioxidant activity in phenolics and composition and metabolism of flavonoids and related compounds in methanol extracts from bulbs of three *Lilium* species. J. Plant Resour Environ..

[62] Lau A.J., Chang T.K.H. (2015). 3-Hydroxyflavone and structural analogueues differentially activate pregnane X receptor: Implication for inflammatory bowel disease. Pharmacol. Res..

[63] Wu Y.B., Zheng L.J., Wu J.G., Chen T.Q., Yi J., Wu J.Z. (2012). Antioxidantt activities of extract and fractions from *Receptaculum nelumbinis* and related flavonol glycosides. Int. J. Mol. Sci..

[64] Büchter C., Ackermann D., Honnen S., Arnold N., Havermann S., Koch K., Wätjen W. (2015). Methylated derivatives of myricetin enhance life span in *Caenorhabditis elegans* dependent in the transcription factor DAF-16. Food Funct..

[65] Tian L.W., Zhanó Y.J., Wang Y.F., Lai C.C., Yang C.R. (2009). Eucalmaidins A-E, (+)-oleuropeic acid derivatives from the fresh leaves of Eucalyptus maiden. J. Nat. Prod..

[66] Grewal A.S., Singh S., Sharma N., Grover (2020). In silico docking studies of some flavonoids against multi pletarget of Alzheimer’s disease. Plant Arch..

[67] Ramezani M., Darbandi N., Khodagholi F., Hashemi A. (2016). Myricetin protects hippocampal CA3 pytamidal neurons and improves learning and memory impairment in rats with Alzheimer’s disease. Neural Regen. Res..

[68] Lau A.J., Politi R., Yang G., Chang T.K.H. (2016). Cell-based and in silico evidence against quercetin and stucturally-related flavonols as activators of vitamin D receptor. J. Steroid Biochem. Mol. Biol..

[69] Hsu Y.L., Liang H.L., Hung C.H., Kuo P.L. (2009). Syringetin, a flavonoid derivatives in grape and wine, induced human osteoblast differentation through bone morphogenetic protein-2/extracellular signal-regulated kinase ½ pathway. Mol. Nutr. Food Res..

[70] Łyko L., Olech M., Nowak R. (2022). LC-ESI-MS/MS characterization of concentrated polyphenolic fractions from *Rhododendron luteum* and their antiinflammatory and antioxidant activities. Molecules.

[71] Neves M., Antunes M., Fernandes W., Campos M.J., Azevedo Z.M., Freitas V., Rocha J.M., Tecelāo C. (2021). Physicochemical and nutritional profile of leaves, flowers, and fruits of the edible halophyte chorão-da-praia (*Carpobrotus edulis*) on Portuguese west shores. Food Biosci..

[72] Cui H.Q., Peng C.Y., Huang Y.Z., Gao Y., Liu J.Q., Zhang R., Shu J.C. (2016). Flavonoids from leaves of *Psidum littora*. Yao Xue Xue Bao.

[73] Beck S., Stengel J. (2016). Mass spectrometric imaging of flavonoid glycosides and biflavonoids in *Ginkgo biloba* L.. Phytochemistry.

[74] Kaur B., Kumar B., Kaur G., Chakaraborty D., Kaur K. (2015). Application of recombinant *Pediococcus acidilactici* BD16 (fcs+/ech+) in malolactic fermentation. Appl. Microbiol. Biotechnol..

[75] Chang W.A., Hung J.Y., Tsai Y.M., Hsu Y.L., Chiang H.H., Chou S.H., Huang M.S., Kuo P.L. (2016). Laricitrin suppresses increased benzo(a)pyrene-induced lung tumor-associated monocyte-derived dendritic cell cancer progression. Onlcol. Lett..

[76] Chang W.A., Hung J.Y., Jian S.F., Lin Y.S., Wu C.Y., Hsu Y.L., Kuo P.L. (2016). Laricitrin ameliorates lung cancer-mediated dendritic cell suppression by inhibiting signal transducer and activator of transcription 3. Oncotarget.

[77] Romani A., Casciano F., Stevanin C., Maietti A., Tedeschi P., Secchiero P., Marchetti N., Voltan R. (2021). Anticancer activity of aqueous extracts from *Asparagus officinalis* L. byproduct on breast cancer cells. Molecules.

[78] Tan K.W., Li Y., Paxton J.W., Birch N.P., Scheepens A. (2013). Identification of novel dietary phytochemicals inhibiting the efflux transporter breast cancer resistance protein (BCRP/ABCG2). Food Chem..

[79] Mattivi F., Guzzon R., Vrhovsek U., Stefanini M., Velasco R. (2006). Metabolite profiling of grape: Flavonols and anthocyanins. J. Agric. Food Chem..

[80] Fioroto C.K.S., da Silva T.B.V., Castilho P.A., Uber T.M., Sá-Nakanishi A.B., Seixas F.A.V., Peralta R.M., Bracht A. (2022). Effects of Ilex paraguariensis beverages on in vivo trigliceride and starch absorbtion in mice. Biocatal. Agric. Biotechnol..

[81] Xie J., Li M.X., Du Z.Z. (2022). Chemical compounds, anti-aging and antibacterial properties of *Rosa rugosa* purple branch. Ind. Crop. Prod..

[82] Kostikova V.A., Zarubaev V.V., Esaulkova I.L., Sinegubova E.O., Kadyrova R.A., Shaldaeva T.M., Veklich T.N., Kuznetsov A.A. (2022). The antiviral, antiradical, and phytochemical potential of dry extracts from *Spiraea hypericifolia*, *S. media*, and *S. salicifolia* (*Rosaceae*). S. Afr. J. Bot..

[83] Gu Q., Duan G., Yu X. (2019). Bioconversion of flavonoid glycosides from *Hippophae rhamnoides* leaves into flavonoid aglycones by *Eurotium amstelodami*. Microorganisms.

[84] Yang Q., Wang Z., Chen X., Guo Z., Wen L., Kan J. (2022). Evaluation of bitter compounds in Zanthoxylum schinifolium Sieb. Et Zucc. by instrumental and sensory analyses. Food Chem..

[85] Shang Z., Li M., Zhang W., Cai S., Hu X., Yi J. (2022). Analysis of phenolic compounds in pickled chayote and their effects on antioxidant activities and cell protection. Food Res. Int..

[86] Yingzhuan Z., Wenjing T., Wenjuan T., Ruochen H., Jue W., Cheng W., Wen L. (2021). Potential antiviral activity of isorhamnetin agains SARS-CoV-2 spike pseudotyped virus in vitro. Drug Dev. Res..

[87] Xiao G., Zeng Z., Jiang J., Xu A., Li S., Li Y., Chen Z., Chen W., Zhang J., Bi X. (2022). Network pharmacology analysis and experimental validation to explore the mechanism of Bushao Tiaozhi capsule (BSTZC) on hyperlipidemia. Sci. Rep..

[88] Vasilakopoulou P.B., Fanarioti E., Tsarouchi M., Kokotou M.G., Dermon C.R., Karathanos V.T., Chiou A. (2022). Polar phenol detection in rat brain: Development and validation of a versatile UHPLC-MS method and application on the brain tissues of corinthian currant (*Vitis vinifera* L., var. Apyrena) fed rats. Food Chem..

[89] Gyeltshen T., Jordan G.J., Smith J.A., Bissember A.C. (2022). Natural products isolation studies of the paleoendemic plant species *Nothofagus gunnii* and *Nothofagus cunninghamii*. Fitoterapia.

[90] Wang Y., Hamburger M., Gueho J., Hostettmann K. (1989). Antimicrobial flavonoids from *Psiadia trinervia* and their methylated and acetylated derivatives. Phytochemistry.

[91] Arciniegas A., Pèrez-Castorena A.L., Melèndez-Aguirre M., Àvila J.G., García-Bores A.M., Villaseñor J.L., Romo de Vivar A. (2018). Chemical composition and antimicrobial activity of *Ageratina deltoidea*. Chem. Biodiv..

[92] Ahn J., Pei Y., Chae H.S., Kim S.H., Kim Y.M., Choi Y.H., Lee J., Chang M., Song Y.S., Rodriguez R. (2018). Spiroketones and a biphenyl analogue from stems and leaves of *Larrea nitida* and their inhibitory activity against IL-6 production. Molecules.

[93] Murata T., Selenge E., Suganuma K., Asai Y., Batkhuu J., Yoshizaki F. (2013). Chromone acyl glucosides and an ayanin glucoside from *Dasiphora parvifolia*. Phytochem. Lett..

[94] Ji H.S., Li H., Mo E.J., Kim U.H., Kim Y.H., Park H.Y., Jeong T.S. (2019). Low-density lipoprotein-antioxidant flavonoids and a phenolic ester from *Plectranthus hadiensis* var. *tomentosus*. Appl. Biol. Chem..

[95] Yamauchi K., Afroze S.H., Mitsunaga T., McCormick T.C., Kuehl T.J., Zawieja D.C., Uddin M.N. (2017). 3,4′,7-O-trimethylquercetin inhibits invasion and migration of ovarian cancer cells. Anticancer Res..

[96] Pick A., Müller H., Mayer R., Haenisch B., Pajeva I.K., Weigt M., Bönisch H., Müller C.E., Wiese M. (2011). Structure-activity relationships of flavonoids as inhibitors of breast cancer resistance protein (BCRP). Bioorg. Med. Chem..

